# Serum biochemical reference interval determination in wild Siberian weasel (*Mustela sibirica*)

**DOI:** 10.17221/102/2022-VETMED

**Published:** 2023-03-28

**Authors:** Minjong Ha, Young Deok Suh, Sohail Ahmed, Do-Na Lee, Jang-Hee Han, Young Ki Kim, Seong-Chan Yeon

**Affiliations:** ^1^College of Veterinary Medicine and Research Institute for Veterinary Science, Seoul National University, Gwanak-gu, Seoul, Republic of Korea; ^2^Seoul Wildlife Center, Seoul National University, Gwanak-gu, Seoul, Republic of Korea; ^3^The Bone Animal Medical Center, Nam-gu, Busan, Republic of Korea

**Keywords:** automated analyser, free-ranging Siberian weasel, serum biochemistry profiles, wildlife conservation, wildlife species

## Abstract

Determining reference intervals (RI) is a valuable asset for assessing the health of wildlife species. This is the first study to establish serum biochemical RIs in Siberian weasels. Forty-two healthy free-ranging Siberian weasels were captured live and brought to Seoul Wildlife Center between June 2021 and August 2022. Blood samples from 42 healthy Siberian weasels of both sexes were used to calculate RIs. An automated analyser was used to perform serum biochemistry profiles. The American Society for Veterinary Clinical Pathology recommendations were used to calculate a nonparametric RI with 90% confidence intervals. The RIs of albumin, total protein, globulin, calcium, glucose, blood urea nitrogen, phosphorus, amylase, cholesterol, alanine aminotransferase, total bilirubin, alkaline phosphatase, creatinine, and creatine kinase were determined. The RIs established in this study will serve as a good starting point for analysing serum biochemical data in Siberian weasels.

Improving the knowledge of animal physiological values is a critical task for wildlife researchers, including the determination of reference intervals (RI). Particularly, serum biochemical indices are crucial for the diagnosis and management of diseases. However, factors such as capture stress, phylogenetic traits, ecology, and the life history of an individual can all have an impact on physiological measurements (Madliger et al. 2018), especially when dealing with wildlife species. To better understand the average, limits, and aberrant values of serum biochemical indices, a large database of wildlife species is required.

The Siberian weasel (*Mustela sibirica*; Pallas, 1773) is a broadly spread small-size musteline with an elongated body. A key characteristic that distinguishes *M. sibirica* is a black mask on its face that surrounds the eyes, a white muzzle and chin, and a monotone yellowish-brown coat (Law 2018; Figure 1). Their native habitats include primary and secondary deciduous, coniferous, and mixed woods; woodlands; open grasslands; and river valleys (Abramov et al. 2016).

**Figure 1:**
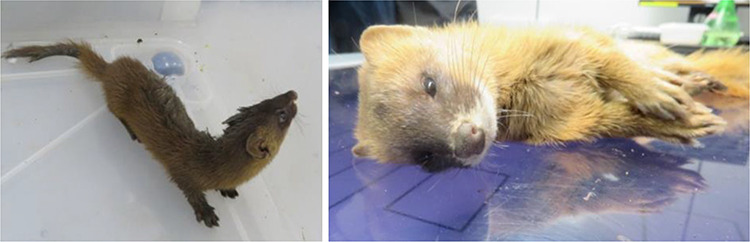
Presence of a nearly completely monotone yellowish-brown coated Siberian weasel (*Mustela sibirica*) (left). The occurrence of a black mask on its face that surrounds the eyes, and a white muzzle and chin (right)

The baseline serum biochemical interval of 42 live-trapped free-ranging Siberian weasels (10 adult males, 11 adult females, 11 juvenile males, and 10 juvenile females) between 2021 and 2022 in Seoul, Republic of Korea (Table 1) was determined using the American Society for Veterinary Clinical Pathology (ASVCP) guidelines for establishing RIs in veterinary species (Friedrichs et al. 2012).

**Table 1:** Number of blood specimens from Siberian weasels used to determine serum biochemistry baseline parameters

Maturity	Male^a^	Female^b^
Adult	10	11
Juvenile	11	10

^a^For males, those weighing more than 395 g were classified as adults; ^b^For females, those weighing more than 300 g were classified as adults

## MATERIAL AND METHODS

### Animals

**Figure 2:**
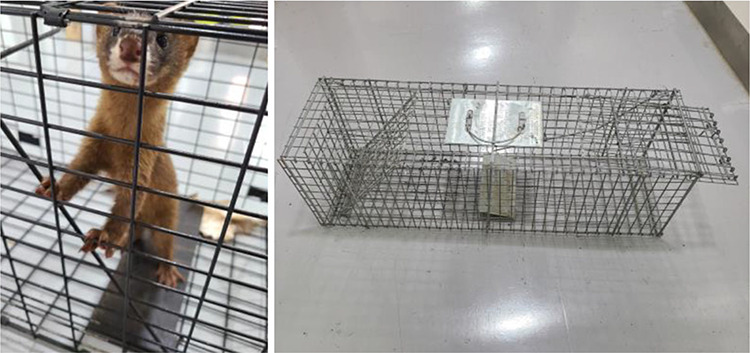
Healthy free-ranging Siberian weasel (*Mustela sibirica*) live captured in Seoul by local trappers (left) with various sizes of wire-walled traps, also known as cage traps (left and right)

Between June 2021 and August 2022, 42 healthy free-ranging Siberian weasels were captured and relocated to Seoul Wildlife Center by local trappers using various sizes of wire-walled traps, often known as cage traps (Figure 2). They were promptly separated in cages upon arrival at the wildlife centre. To alleviate the stress of transport, a warm, dark, and quiet room with food and water was supplied for at least 24 h before blood collection. To satisfy their daily requirements, one-day-old chicks and quails were fed. The wildlife centre’s vets examined the Siberian weasels’ health and determined that there were no unusual findings. As a result, all the animals were released a week following stabilization.

Body weight was utilised as a criterion to estimate the age of the animals. Males weighing more than 395 g and females weighing more than 300 g were considered adults (Heptner et al. 2001).

All experiments were conducted in accordance with the ARRIVE guidelines (Animal Research: Reporting of *In Vivo* Experiments) developed for the ethical use and reporting of laboratory animals. A completed ARRIVE guidelines checklist is included in Checklist [see electronic supplementary materials (ESM)]. All animal experiments were approved by the Institutional Animal Care and Use Committee of Seoul National University (SNU-IACUC; Approval No.: SNU-221101-1). All protocols involving animals were conducted in accordance with the following South Korean legislations: Animal Protection Act, Laboratory Animal Act, and Wildlife Protection and Management Act.

### Anaesthesia

For immobilization of the animal, a mixture of alfaxalone (5 mg/kg, Alfaxan Multidose; Jurox, Rutherford, NSW, Australia) and medetomidine (20 μg/kg, Tomidine; Provet, Istanbul, Turkiye) was administered by intramuscular injection. During the procedure, supplemental oxygen was provided via a face mask at a flow rate of 2 l/min. During a 15-min anaesthetic procedure, a physical examination and blood collection were done.

### Specimen handling

**Figure 3:**
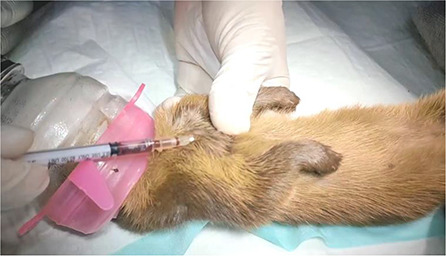
Blood samples (0.7–1.0 ml) were taken from the cranial *vena cava* of anaesthetised Siberian weasels (*Mustela sibirica*) with a 1-ml syringe and 26-gauge needle

Blood (0.7–1.0 ml) was drawn from the cranial *vena cava* of sedated Siberian weasels using a 1 ml syringe and a 26-gauge needle, as in domestic ferrets (Queensberry and de Matos 2020). A representative image is presented in Figure 3. For serum biochemistry, blood samples were placed in serum separator vacutainer tubes. After allowing blood samples for serum biochemistry tests to coagulate for 30 min, they were centrifuged for 10 min at 1 300 *g*. Centrifugation was used to separate the serum, which was then transferred into microtubes. Samples with haemolysis or lipemia were excluded to prevent preanalytical errors.

### Sample analysis

Using an automated analyser (Pointcare V2; Mnchip, Tianjin, P.R. China), serum biochemistry results, including albumin (ALB; non-enzymatic colour assay), total protein (TP; non-enzymatic colour assay), globulin (GLO), albumin-to-globulin ratio (A/G), calcium (Ca; non-enzymatic colour assay), glucose (GLU; UV-test), blood urea nitrogen (BUN; enzymatic method), phosphorous (P; enzymatic method), amylase (AMY; enzymatic method), cholesterol (CHOL; enzymatic method), alanine aminotransferase (ALT; UV-test), total bilirubin (TBIL; enzymatic method), alkaline phosphatase (ALP; non-enzymatic colour assay), creatinine (CRE; enzymatic method), and creatine kinase (CK) were obtained. The principles in the procedure referred to the operation manual of the manufacturer.

### Statistical analysis

RIs were calculated in accordance with the ASVCP’s (American Society for Veterinary Clinical Pathology) recommendations (Friedrichs et al. 2012). The animals were divided into subgroups based on their age and sex. Descriptive statistics and RIs with confidence intervals were calibrated using spreadsheet software (Excel v2303; Microsoft Corp., USA) with the Reference Value Advisor (v2.1) freeware and Prism v9 (GraphPad Software, USA). Outliers were deleted, if found, according to Tukey or Dixon. Statistical normality was evaluated using the Anderson-Darling test or D’Agostino & Pearson test. According to the sample size, RIs were determined using a nonparametric method. The 90% confidence intervals of upper and lower nonparametric RIs were determined using the bootstrap method. The Kruskal-Wallis test was performed to compare the relevance of the parameters with sex and age. Group differences among species were analysed with Student’s *t*-test and one-way analysis of variance (ANOVA) followed by Duncan’s test if significant. The *P*-value < 0.05 was considered significant in statistical analysis.

## RESULTS

This study involved 42 healthy free-ranging Siberian weasels. For statistical analysis, the samples were divided into four subgroups: 10 adult males, 11 adult females, 11 juvenile males, and 10 juvenile females.

RI (means) for serum biochemistry in this study (Table 2) were established as albumin 9.90–40.90 g/l (31.50), TP 16.30–79.30 g/l (56.90), globulin 5.70–39.50 g/l (25.40), albumin-to-globulin ratio 0.72–2.29 (1.28), calcium 1.58–3.13 mmol/l (2.52), glucose 1.65–33.41 mmol/l (8.11), BUN 4.79–30.22 mmol/l (13.04), phosphorus 1.46–6.59 mmol/l (2.64), amylase 0.19–2.78 μkat/l (1.02), cholesterol 1.49–10.51 mmol/l (6.57), alanine aminotransferase 1.02–3.93 μkat/l (2.01), TBIL 2.05–16.42 μmol/l (4.28), alkaline phosphatase 0.35–4.96 μkat/l (1.64), creatinine 27.40–135.25 μmol/l (53.93), and CK 1.8–11.29 μkat/l (6.23).

**Table 2:** Serum biochemistry values for 42 healthy, free-ranging, and live-trapped Siberian weasels (*Mustela sibirica*)

Parameters	Unit	Mean	Median	SD	RI	90% CI for RI LL	90% CI for RI UL
ALB	g/l	31.50	32.50	6.50	9.90–40.90	9.00–22.30	39.90–41.00
TP	g/l	56.90	57.60	10.90	16.30–79.30	14.00–45.00	70.00–80.00
GLO	g/l	25.40	25.40	5.90	5.70–39.50	5.00–15.40	31.90–40.00
A/G	–	1.28	1.20	0.32	0.72–2.29	0.70–0.91	1.79–2.30
Ca	mmol/l	2.52	2.57	0.34	1.58–3.13	1.57–1.82	2.89–3.14
GLU	mmol/l	8.11	7.47	5.15	1.65–33.41	1.61–2.55	12.50–34.97
BUN	mmol/l	13.04	12.09	5.73	4.79–30.22	4.79–5.79	22.98–30.39
P	mmol/l	2.64	2.58	0.97	1.46–6.59	1.45–1.59	4.25–6.75
AMY	μkat/l	1.02	0.95	0.60	0.19–2.78	0.18–0.32	2.32–2.81
CHOL	mmol/l	6.57	6.58	1.95	1.49–10.51	1.37–3.41	9.26–10.60
ALT	μkat/l	2.01	1.98	0.69	1.02–3.93	1.01–1.18	3.07–3.95
TBIL	μmol/l	4.28	4.10	2.90	2.05–16.42	2.05–2.22	5.13–16.59
ALP	μkat/l	1.64	1.31	1.12	0.35–4.96	0.33–0.60	3.25–4.98
CRE	μmol/l	53.93	48.62	22.98	27.40–135.25	27.40–28.29	85.75–138.79
CK	μkat/l	6.23	5.33	2.53	1.80–11.29	1.78–2.28	9.27–11.35

A/G = albumin-to-globulin ratio; ALB = albumin; ALP = alkaline phosphatase; ALT = alanine transaminase; AMY = amylase; BUN = blood urea nitrogen; Ca = calcium; CHOL = cholesterol; CI = confidence interval; CK = creatine kinase; CRE = creatinine; GLO = globulin; GLU = glucose; LL = lower limit; P = phosphorus; RI = reference interval; SD = standard deviation; TBIL = total bilirubin; TP = total protein; UL = upper limit

The following serum biochemistry values were shown to have statistical significance in terms of sex and/or age: calcium, amylase, TBIL, alkaline phosphatase, and creatinine (Table 3).

**Table 3:** Serum biochemistry values (mean ± SD) for 42 healthy, free-living, and live-trapped Siberian weasels (*Mustela sibirica*)

Parameter	Unit	Male (adult)	Female (adult)	Male (juvenile)	Female (juvenile)
ALB	g/l	33.60 ± 4.80	32.70 ± 5.80	27.70 ± 8.80	31.90 ± 5.50
TP	g/l	60.50 ± 6.60	57.60 ± 9.00	51.40 ± 15.10	57.60 ± 11.10
GLO	g/l	26.90 ± 3.10	24.90 ± 4.40	23.70 ± 8.40	25.70 ± 6.80
A/G	–	1.29 ± 0.34	1.28 ± 0.20	1.28 ± 0.49	1.29 ± 0.34
Ca*	mmol/l	2.64 ± 0.21	2.68 ± 0.18	2.27 ± 0.48	2.50 ± 0.31
GLU	mmol/l	8.12 ± 2.60	9.01 ± 2.75	9.04 ± 9.64	6.43 ± 2.62
BUN	mmol/l	13.59 ± 7.01	10.78 ± 2.89	13.53 ± 5.29	14.10 ± 6.77
P	mmol/l	2.36 ± 0.55	2.44 ± 0.56	2.89 ± 1.60	2.86 ± 0.85
AMY*	μkat/l	0.90 ± 0.28	1.04 ± 0.44	0.87 ± 0.93	1.28 ± 0.57
CHOL	mmol/l	6.78 ± 1.88	7.35 ± 1.47	5.42 ± 2.05	6.70 ± 2.07
ALT	μkat/l	2.05 ± 0.85	2.17 ± 0.64	2.07 ± 0.80	1.75 ± 0.45
TBIL*	μmol/l	3.25 ± 0.86	3.93 ± 1.03	4.45 ± 3.93	5.64 ± 3.76
ALP*	μkat/l	1.36 ± 0.53	2.16 ± 1.34	1.18 ± 1.27	1.88 ± 1.10
CRE*	μmol/l	58.35 ± 30.94	43.32 ± 14.14	70.72 ± 15.91	42.43 ± 13.26
CK	μkat/l	5.87 ± 2.42	5.76 ± 2.84	6.58 ± 2.59	6.70 ± 2.52

*Statistically significant (*P* < 0.05); The Kruskal–Wallis test was performed to compare the relevance among subgroups

A/G = albumin-to-globulin ratio; ALB = albumin; ALP = alkaline phosphatase; ALT = alanine transaminase; AMY = amylase; BUN = blood urea nitrogen; Ca = calcium; CHOL = cholesterol; CK = creatine kinase; CRE = creatinine; GLO = globulin; GLU = glucose; P = phosphorus; SD = standard deviation; TBIL = total bilirubin; TP = total protein

## DISCUSSION

Forty-two Siberian weasels were used in this study. Their blood biochemistry data were used to calculate the RI, and statistical assessments of the parameters’ relevance with regard to sex and age variables were completed.

In this study, serum calcium concentrations were higher in adults than in juveniles, which is consistent with earlier research on several species (Parhon and Werner 1932; Watchorn 1933). It is possible that it is related to vitamin D deficiency and seasonal fluctuations in their intake of this vitamin (Watchorn 1933).

Females showed higher amylase activities than males in agreement with earlier data obtained in humans and red howler monkeys (Segawa et al. 1989; Vie et al. 1998). According to Koop (1984), the determinants of serum amylase are the amount of serum amylase produced, degree of leakage from the pancreas or salivary glands into the blood, urinary function, and molecular weight of amylase.

TBIL levels were greater in juveniles than in adults in this study. This finding is consistent with prior studies conducted on Arabian mares (Gurgoze and Icen 2010) and neonatal rats and humans (Johnson et al. 1983). It could be the result of foetal RBC destruction in the liver and spleen’s mononuclear phagocyte system (Johnson et al. 1983).

Serum alkaline phosphatase activity appears to be influenced by a number of factors, including age, sex, nutrition, hunger, and environmental changes (Durak et al. 2015). Oestradiol decreases serum alkaline phosphatase activity, whereas testosterone propionate raises it (Hoffmann et al. 1994). On the contrary, in this study on Siberian weasel’s alkaline phosphatase levels were higher in females. Further studies are required to verify the association between sex and serum alkaline phosphatase activity in Siberian weasels.

It is well known that creatinine values in male humans are 30% higher than in females, and urine creatinine excretion is correlated to muscle mass across a wide range of animal species, lending credence to this relationship (Siegel and Walton 2020). The results of this study on Siberian weasels correspond to the above-mentioned data showing higher creatinine levels in males.

As there are no baseline measurements for Siberian weasels (*Mustela sibirica*), serum biochemistry values in this study were compared with domestic ferrets (*Mustela putorius*; Ohwada and Katahira 1993) and minks (*Mustela vison*; Weiss et al. 1994), two species with close phylogenetic relationships (Table 4). There were significant differences in albumin, albumin-to-globulin ratio, and alanine aminotransferase levels between Siberian weasels and domestic ferrets. Siberian weasels and minks have significantly different BUN, alanine aminotransferase, calcium, and phosphorus levels. All three species had significantly different alanine aminotransferase activities.

**Table 4:** Comparison of serum biochemical values (mean ± SD) among Siberian weasel, domestic ferret, and mink

Parameter	Unit	Current study on Siberian weasel (*Mustela sibirica*)	Domestic ferret (*Mustela putorius*)^a^	Mink (*Mustela vison*)^b^
ALB	g/l	31.50 ± 6.50^c^	8.50 ± 1.80^d^	29.90 ± 0.10^c^
TP	g/l	56.90 ± 10.90	52.80 ± 0.90	60.20 ± 1.10
GLO	g/l	25.40 ± 5.90	–	–
A/G	–	1.28 ± 0.32^c^	2.28 ± 0.18^d^	–
Ca	mmol/l	2.52 ± 0.34^c^	2.86 ± 0.38^c^	2.37 ± 0.01^e^
GLU	mmol/l	8.11 ± 5.15	–	6.93 ± 0.07
BUN	mmol/l	13.04 ± 5.73^c^	8.24 ± 0.72^c^	5.61 ± 0.25^e^
P	mmol/l	2.64 ± 0.97^c^	1.95 ± 0.36^c^	1.69 ± 0.02^e^
AMY	μkat/l	1.02 ± 0.60	–	–
CHOL	mmol/l	6.57 ± 1.95	–	–
ALT	μkat/l	2.01 ± 0.69^c^	0.65 ± 0.30^d^	1.26 ± 0.10^e^
TBIL	μmol/l	4.28 ± 2.90	4.10 ± 0.51	–
ALP	μkat/l	1.64 ± 1.12	0.05 ± 0.00	–
CRE	μmol/l	53.93 ± 22.98	73.37 ± 7.07	59.23 ± 5.30
CK	μkat/l	6.23 ± 2.53	–	–

^a^Data from Ohwada and Katahira (1993); ^b^Data from Weiss et al. (1994); ^c–e^Data were analysed by Student’s *t*-test and one-way analysis of variance (ANOVA) followed by Duncan’s test if significant. Different letters in the same column indicate significant differences (*P* < 0.05)

A/G = albumin-to-globulin ratio; ALB = albumin; ALP = alkaline phosphatase; ALT = alanine transaminase; AMY = amylase; BUN = blood urea nitrogen; Ca = calcium; CHOL = cholesterol; CK = creatine kinase; CRE = creatinine; GLO = globulin; GLU = glucose; P = phosphorus; TBIL = total bilirubin; TP = total protein

There may be some possible limitations in this study. The Clinical and Laboratory Standards Institute (Horowitz et al. 2008) recommends a minimum sample size of 120 for RI determination. Even though it was attempted to close the gap using statistical estimation methodology, there may be a difference when research is conducted on larger sample sizes. Furthermore, this research is being conducted on wild animals. Several variables, including phylogenetic features of subpopulations, living habitat, and life history, can influence physiological patterns in this setting. Stress variables such as transit or capture stress, seasonal fluctuations, nutritional conditions, and reproductive state can all have an effect on physiological values. In addition, although there are statistical differences with respect to sex and age in this study, the clinical significance of the parameters could not be assessed. In this preliminary study of 42 samples, there was no indication of clinical difference; however, future research will need to evaluate the clinical significance of the variables with long-term trends using a larger sample size. Also, it has to be noted that the difference between analytes when comparing species with close phylogenetic relationships may be attributable to methodological differences and not species differences. Still, serum biochemical values are crucial for diagnosis and health management, and analysing these data in the field for wildlife species is a vital step. As a result, the purpose of this study was to collect baseline serum biochemistry data for wild Siberian weasels.

In conclusion, it could be stated that the serum biochemical profile of healthy, free-ranging Siberian weasels was determined in this study. Using the baseline measures established in this study, the health of the Siberian weasel population could be evaluated. It might also become a basis for future studies to protect wildlife species.
